# Sudden death of an egg donor during oocyte retrieval due to ovarian hyperstimulation syndrome

**DOI:** 10.4322/acr.2021.385

**Published:** 2022-05-27

**Authors:** Swati Tyagi, Asit Ranjan Mridha, Chittaranjan Behera

**Affiliations:** 1 All India Institute of Medical Sciences, Department of Forensic Medicine & Toxicology, New Delhi, Delhi, India; 2 All India Institute of Medical Sciences, Department of Pathology, New Delhi, Delhi, India

**Keywords:** Oocyte Donation, Pulmonary Edema, Death, Sudden, Ovarian Hyperstimulation Syndrome

## Abstract

Ovarian Hyperstimulation Syndrome (OHSS) is uncommon among oocyte donors during in vitro fertilization (IVF) procedure and is rarely associated with death. We report a case of a 23-year-old oocyte donor who suddenly died on the operation table during oocyte retrieval. She had no risk factors in her menstrual history, laboratory, or clinical parameters. The antagonist cycle, triggered with the GnRH agonist protocol, was carried out. The cause of death at autopsy was attributed to respiratory failure due to acute massive pulmonary edema, which developed due to the complication of OHSS. Only a few autopsy cases associated with OHSS have been published, but, as far as we know, no clinical or autopsy cases of sudden death caused by OHSS have been reported.

## INTRODUCTION

Ovarian hyperstimulation syndrome (OHSS) is a rare and severe complication during controlled ovarian stimulation (COS) due to an exaggerated response to ovulation induction that is presumed to induce secretion of ovarian vasoactive-angiogenic substances like estrogen, prostaglandin gonadotropin, histamine, cytokines, prolactin, renin-angiotensin, and aldosterone. The vasoactive substances increase the capillary permeability and induce fluid accumulation in the extravascular space.^1^ However, the role of all of these causative agents in initiating the cascade of clinical events that lead to OHSS remains unknown.

The primary risk factors for OHSS are young age, low body mass index, polycystic ovarian syndrome (PCOS), and previous episodes of OHSS. Secondary risk factors include serum estradiol (E2) levels greater than 3000-4000 pg/mL in IVF cycles and greater than 1500-2000 pg/mL in COS cycles, follicle numbers greater than 20-25 in both ovaries, a large number of oocyte retrievals, stimulation procedures and agents used, and hCG (human Chorionic Gonadotropin) administration for luteal support and pregnancy.^1^

According to World Health Organization (WHO), the incidence of severe OHSS is 0.2-1% of all stimulation cycles in assisted reproduction.^2^ Selter et al.^3^ revealed that patients with common comorbidities have a higher risk of life-threatening complications [deep vein thrombosis/pulmonary embolism (2.2%), acute renal failure; acute respiratory distress syndrome (0.9%), and invasive procedures like endotracheal intubation used to manage certain life-threatening complications (0.5%)], which occurred in 4.4% of OHSS patients.

Severe OHSS is a rare event in an egg donor. Sauer^4^ observed that out of 1000 egg donors, 0.7% of cases developed OHSS, and only three cases (0.3%) had severe OHSS. Maxwell et al.^5^ also found a similar incidence (0.7%) of OHSS in 886 egg donors and no severe OHSS in their donors.

OHSS is graded according to severity (mild, moderate, and severe).^6^ Grade 1(mild) OHSS is characterized by fluid accumulation, as evidenced by weight gain and abdominal discomfort, and distension. On ultrasonography, multicystic ovarian enlargement with a diameter higher than 5cm is also seen. Grade 2 (moderate) OHSS is characterized by bloating, and abdominal distension that accompanies nausea and vomiting. Ovarian enlargement and abdominal distension are more pronounced, resulting in increased discomfort and dyspnea. Ascites can be detected by ultrasound. In Grade 3(severe) OHSS, there is clinical evidence of intravascular volume contraction, severe expansion of third space, severe hemoconcentration, development of hepatorenal failure, and intravascular thrombosis.^6^ Death is reported in grade 3 OHSS undergoing IVF due to cerebrovascular thrombosis, renal failure, acute pulmonary edema or cardiac tamponade resulting from pericardial effusion.^6^^-^ 11 However, only a few autopsy case reports are available. In these cases, the death occurred after a few days of completion of the egg donation process.^8^^-^ 11We report a sudden death due to OHSS that happened during oocyte retrieval in an anonymous egg donor.

### Case Report

A 23-year-old woman died during an egg donation procedure at a fertility clinic, with a cardiac arrest as the clinical cause of death. She had been married for seven years and has a four-year-old daughter born via normal childbirth. Her menarche happened at the age of 13, and her cycles were regular, occurring every 28 to 30 days. There was no history of abortion/pregnancy loss, diabetes, hypertension, asthma, allergy/drug allergy, thyroid disorder, epilepsy, tuberculosis and cardiac disorders, tobacco, alcohol, and illicit drug use. Family history was unremarkable. Her weight was 58 kg, her height was 148 cm (BMI-26.5), her blood pressure was 120/80 mmHg, and her pulse rate was 80 beats per minute when she was admitted for the egg donation. Her heart and breath sounds were normal. Laboratory results revealed hemoglobin level 12.0 g/dL (RR:12-15 g/dL), random blood sugar 81mg/dL (RR: 70**-**140mg/dL), SGPT 16 U/L(RR:6-55 U/L), serum creatinine 0.74 mg/dL(RR:0.57-1.11mg/dL), prolactin 8.37 ng/ml(RR:5.18-26.53ng/ml), TSH 1.45 mU/L (RR: 0.35-5.50 mU/L), and antimullerian hormone 4.69ng/ml(RR:1.66-9.49 ng/ml). Lipid profiles were normal. Serum serology for HIV, HBsAg, HCV, VDRL, and Rubella IgM was non-reactive. Serum IgG test for rubella showed an adequate immune response. A baseline transvaginal ultrasonography showed normal uterus and ovaries without any features of polycystic ovaries. There was not any risk factor for coronary artery disease. The clinical history, physical examination, and test results indicated that the patient was a healthy young woman.

After taking her informed consent, an antagonist ovarian stimulation protocol was scheduled. She received oral contraceptives during her menstruation. From the 6^th^ to 8^th^ day of the menstrual cycle, recombinant FSH (rFSH 300 IU s/c, daily) was given. From the 9^th^ to 14^th^ day of the menstrual cycle, HMG 300 IU IM was given every day, and on the 15th day of her menstrual cycle, a dose of HMG 75 IU IM was administered. On the 11^th^ day of the menstrual cycle, she began taking a GnRH antagonist (0.25 IU) and continued until the 15^th^ day of the menstrual cycle. Serum E2 and P4 levels were measured on the 15^th^ day of the menstrual cycle and determined to be 2048 pg/ml and 0.89 ng/ml, respectively. On the same day, ultrasonography showed five follicles in the right ovary with a 17-18 mm diameter and five follicles in the left ovary with 17-19 mm. Triggering was done on the same day (the 15^th^ day of the menstrual cycle) at 10.30pm with decapeptyl 0.2mg s/c (GnRH agonist). Oocyte aspiration through transvaginal ultrasound was planned.

On the morning of the 17th day of the menstrual cycle, a pre-anesthetic check-up revealed no risk factors. Her blood pressure was 120/80 mm Hg. Her pulse rate was 86 beats per minute. Her arterial oxygen saturation was 100%. She was given 1L of Ringer's lactate I.V., followed by 500 ml I.V. slowly as maintenance. Pyrolate and fentanyl were given as premedication. Injection midazolam 1mg was given intravenously. Injection Dynapar 75mg I.V was given slowly. Propofol 120 mg intravenously was used to induce anesthesia, then maintained with sevoflurane. At the start of anesthesia, her blood pressure was 110/68 mmHg, her heart rate was 97/minute, and her SPO2 was 100%. An ECG showed normal sinus rhythm. An ultrasound-guided aspiration of follicles started in the right ovary after confirming no vasculature in between. According to anaesthetic information, after the ovum was picked up from the right ovary, the patient had hypotension (blood pressure decreased from 110/68 mmHg to 70/40 mmHg), bradycardia (heart rate dropped from 97 to 50 per minute), arterial oxygen saturation (SAO2) dropped from 100 to 80%, and the ECG showed bradyarrhythmia. The procedure was immediately abandoned. On auscultation, the heartbeat could not be heard. Air entry was normal. Per vagina examination lacked to detect bleeding. A trans-vaginal ultrasound also showed no intrapelvic or intrabdominal bleeding. Cardiopulmonary resuscitation (CPR) was started. The patient was intubated with a 7.0 mm cuffed endotracheal tube. Position-conformed breaths were given at a rate of 10-12 per minute. Pharmacologic support, including atropine and adrenaline, was given. One 200J DC shock was given. However, her ECG rhythm remained asystole throughout. Unfortunately, she could not be saved, and an autopsy was performed because she died suddenly and unexpectedly.

### Autopsy Findings

**Figure 1:**
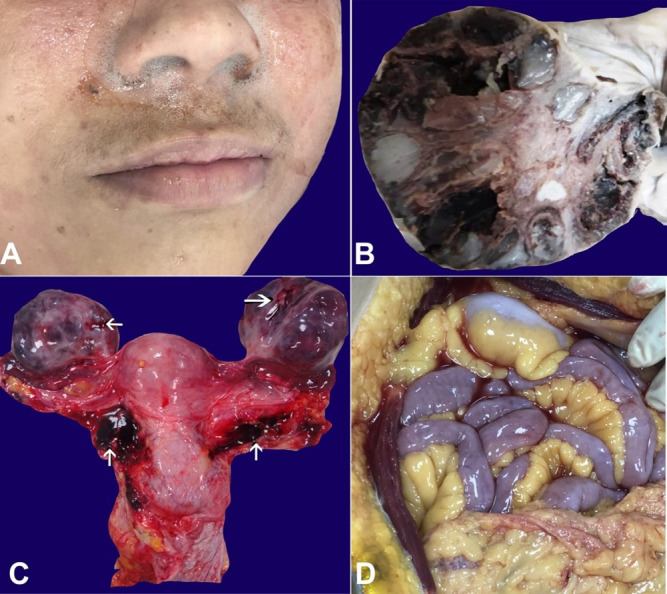
Gross view of: **A –** corpse’s face showing froth coming out from nostrils; **B –** ovary - with multiple enlarged follicles; **C –** uterus and adnexa with multiple puncture marks on ovaries and hematoma in adnexal tissue (arrows); **D –** peritoneal cavity with fluid.

The deceased was a young woman of average build and stature. Rigor mortis was present all over the body. Postmortem staining was present on the back of the body, sparing areas of contact flattening and was fixed. Fine pinkish froth oozed out from the nostrils (Figure 1A).

The uterus was normal and bilateral ovaries were enlarged. The right and left ovaries weighed 95 g (RR:3.67-6.65g) and 105 g (RR:3.10-6.80g), respectively, and were sized 90 × 45 × 30 mm and 75 × 35 × 30 mm (each ovary measures about 35x25x18mm). Multiple puncture marks were present in both ovaries. Multiple enlarged follicles (9 to10) were visible on the cut surfaces of both ovaries; a few of them were hemorrhagic (Figure 1B). Multiple puncture marks were also present in the lateral walls of the upper vagina and bilateral adnexal tissue with clotted blood (Figure 1C). Uterovesical pouch contained about 60-70 cc of hemorrhagic fluid. The peritoneal cavity contained 150-200 ml of mixed blood fluid (Figure 1D).

The pleural cavities contained about 100-120 mL of straw-colored fluid each. The lungs were edematous, with the right lung weighing 460 g (RR: 280-500 g) and the left lung weighing 360 g (RR:240-340 g). There was no evidence of pulmonary infarction or pulmonary emboli. Both adrenal glands were grossly unremarkable. The remaining internal organs were grossly unremarkable.

**Figure 2:**
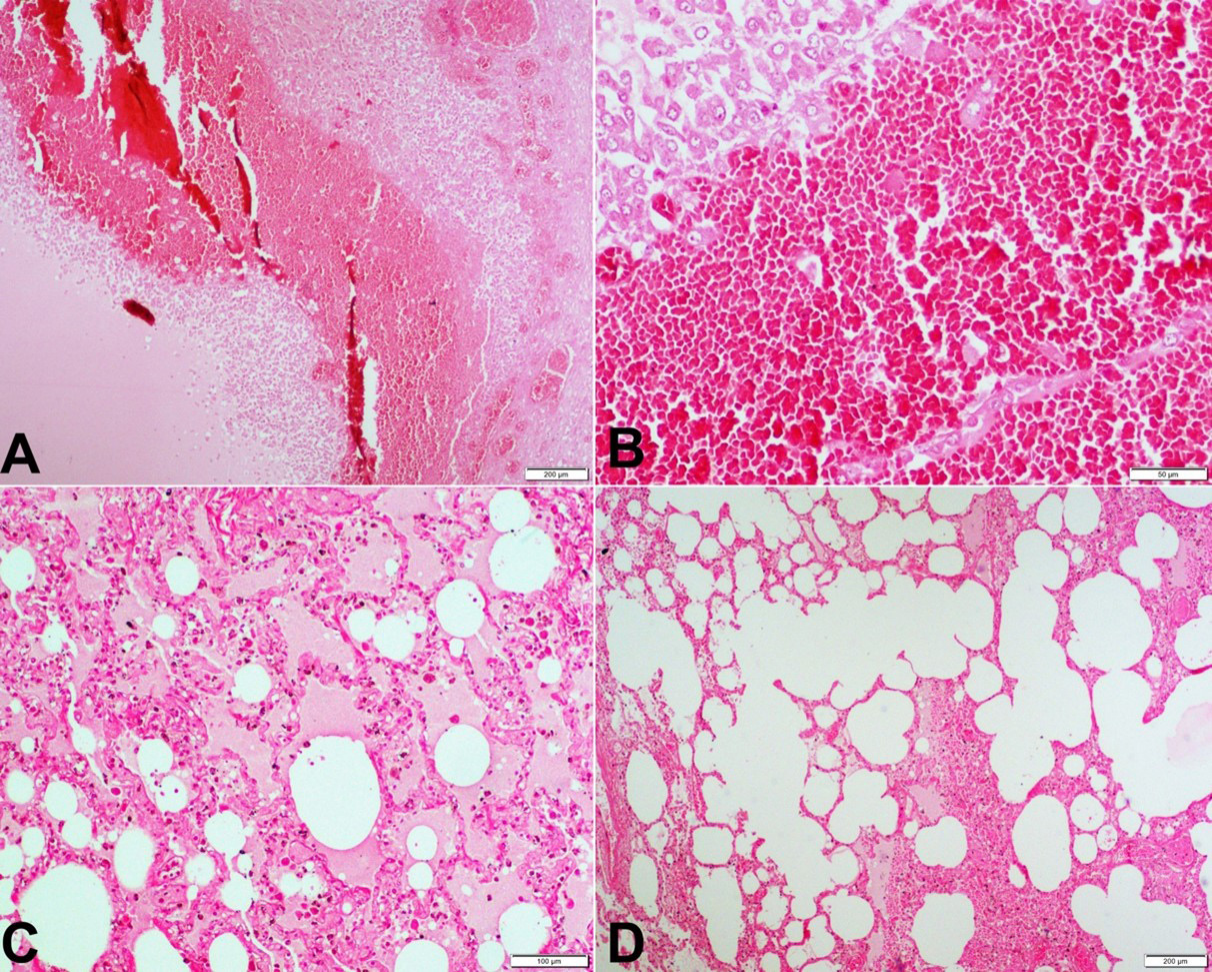
Photomicrographs of: **A –** marked congestion of ovarian microvasculature (HE x40); **B –** ovary with hemorrhage within parenchyma (HE x200); **C –** lungs - showing intra-alveolar edema (HE x100); **D –** lungs with hyper-inflated alveoli with disrupted inter-alveolar septae suggesting air trapping (HE x40).

Microscopic examination of both ovaries showed marked vascular congestion and hemorrhage in the wall of ovarian follicles (Figures 2A and 2B). Intrafollicular hemorrhage was also noted.

Sections from bilateral lungs showed intra-alveolar edema (Figure 2C) and hyper-inflated alveoli with disrupted inter-alveolar septae that suggested air trapping (Figure 2D). The heart, brain, liver, and kidneys were unremarkable.

According to toxicological tests, there were no poisons or drugs found in the viscera, urine sample, or injection site samples. The cause of death was attributed to acute pulmonary edema consequent to ovarian hyperstimulation syndrome.

## DISCUSSION

In artificial reproductive technique (ART), many complications can happen during or following oocyte retrieval, including ovarian hyperstimulation syndrome, ovarian torsion, infection, ovarian cyst ruptured, adverse reaction to intravenous anesthesia, intrabdominal bleeding after aspiration, bladder atony, and hematuria.^4^^,^ 5 In the present case, a healthy young patient suddenly died during oocyte retrieval. At autopsy, characteristic findings of OHSS such as ascites, pleural effusion, acute pulmonary edema, ovarian enlargement with multiple cysts, and features of ovarian stimulation were observed. In view of sudden death, we have considered other possible causes of death like myocardial infarction, pulmonary thromboembolism, pheochromocytoma crisis, and anesthesia complications. Gross and histological examination rule out pulmonary thromboembolism. Gross pathology of the adrenal glands did not reveal any tumor, and there was no mention of any clinical signs of pheochromocytoma in the patient’s clinical record; hence pheochromocytoma crisis is ruled out. The victim was a healthy young female without any risk for cardiovascular disease; hence myocardial infarction is unlikely. Cases have been reported in which myocardial infarction was associated with OHSS.^12^^-^ 15 However, no fatal case has been reported yet. Wang et al.^16^ reported the sudden death of a 29-year-old woman due to severe ovarian hyperstimulation syndrome in the aftermath of assisted reproduction technology and ovarian puncture. At autopsy, turbid effusions of pleural and peritoneal cavities, abnormal ovarian enlargement, and duskiness of multiple organ surfaces were observed. Microscopic examination revealed edema and hemorrhage in follicles of both ovaries, thrombosis within the myocardial matrix, and massive pulmonary edema.

Ovarian hyperstimulation syndrome is believed to stem from exogenous human chorionic gonadotropin administration, which causes the development of multiple ovarian follicles that release large amounts of vasoactive/angiogenic mediators. The ovarian hyperstimulation syndrome is just one of the elements of the spectrum of “capillary leak syndrome”.^17^ In severe OHSS, there is increased capillary leak with massive transudation of protein-rich fluid from the vascular space into the peritoneal, pericardial, and pleural cavities and alveoli resulting in hypovolemia.

In our case, the cause of death was attributed to acute pulmonary edema as the complication of OHSS. In this case, acute pulmonary edema developed as a complication of OHSS, a low-pressure edema. Acute pulmonary edema led to cardiac arrest due to hypoxia. She was not successfully resuscitated. In the present case, antagonist protocol was followed for ovarian stimulation and triggered by a gonadotropin-releasing hormone agonist. GnRHa trigger is currently the best existing tool for preventing OHSS while maintaining acceptable reproductive outcomes.^18^ In the last decade, the substitution of the human chorionic gonadotropin trigger with the gonadotropin-releasing hormone agonist trigger has decreased the incidence of OHSS but did not eliminate it.^19^ The risk factors for the development of OHSS in our case were young age (23 years), and high serum E2 level (2048 pg/mL) in the controlled ovarian stimulation cycle.^1^ In younger women, there is a greater ovarian response for the development of OHSS than the older woman.

In our case, OHSS developed within 48 hours after triggering with GnRHa. Usually, early OHSS occurs within 10 days after the ovulation, triggering hCG injection and is associated with higher serum estradiol levels and a larger number of follicles. However, at autopsy, about 9 to 10 ovarian follicles were observed in each ovary, in our case. Late OHSS (after 10 days of administration of hCG) is more closely linked to the endogenous hCG, produced during pregnancy.^20^^,^ 21 Mahajan et al.^22^ reported a case of early-onset severe ovarian hyperstimulation syndrome (OHSS) presenting with oliguria in an antagonist cycle triggered with GnRH agonist and a freeze-all approach. However, this case was managed in an ICU with albumin and diuretics.

In our case, there were multiple puncture marks on both ovaries and vaginal walls, leading to hematoma in the adnexa and uterovesical pouch. The role of trauma due to the procedure to the already hyperstimulated ovaries for the production of OHSS is unknown. Saha et al.,^23^^,^ 24 however, reported that caution should be taken with pelvic examinations to minimize the risk of enlarged ovaries trauma. Pooniya et al.^11^ also reported OHSS in an egg donor with multiple injuries present on the ovary and vaginal wall.

## CONCLUSION

Recently, egg donation cases have been on the rise due to the high demand for IVF. The prospective oocyte donors should be adequately counseled about the risks related to egg donation. Risk factors like high serum E2 levels must be assessed, and precautions should be taken. During the procedure, caution should also be taken not to injure the enlarged ovaries. Patients at risk of OHSS should be closely monitored following ovum pickup, even when an agonist trigger has been given for early detection and management.^21^ In case of severe OHSS, proper evaluation of the patient and intensive care are required.
